# Supplementary figures and images for: Discovery of Salidroside as a Novel Non-Coding RNA Modulator to Delay Cellular Senescence and Promote BK-Dependent Apoptosis in Cerebrovascular Smooth Muscle Cells of Simulated Microgravity Rats
Source: Int J Mol Sci. 2023 Sep 26;24(19):14531. doi: 10.3390/ijms241914531 (PMC10572139; doi:10.3390/ijms241914531)

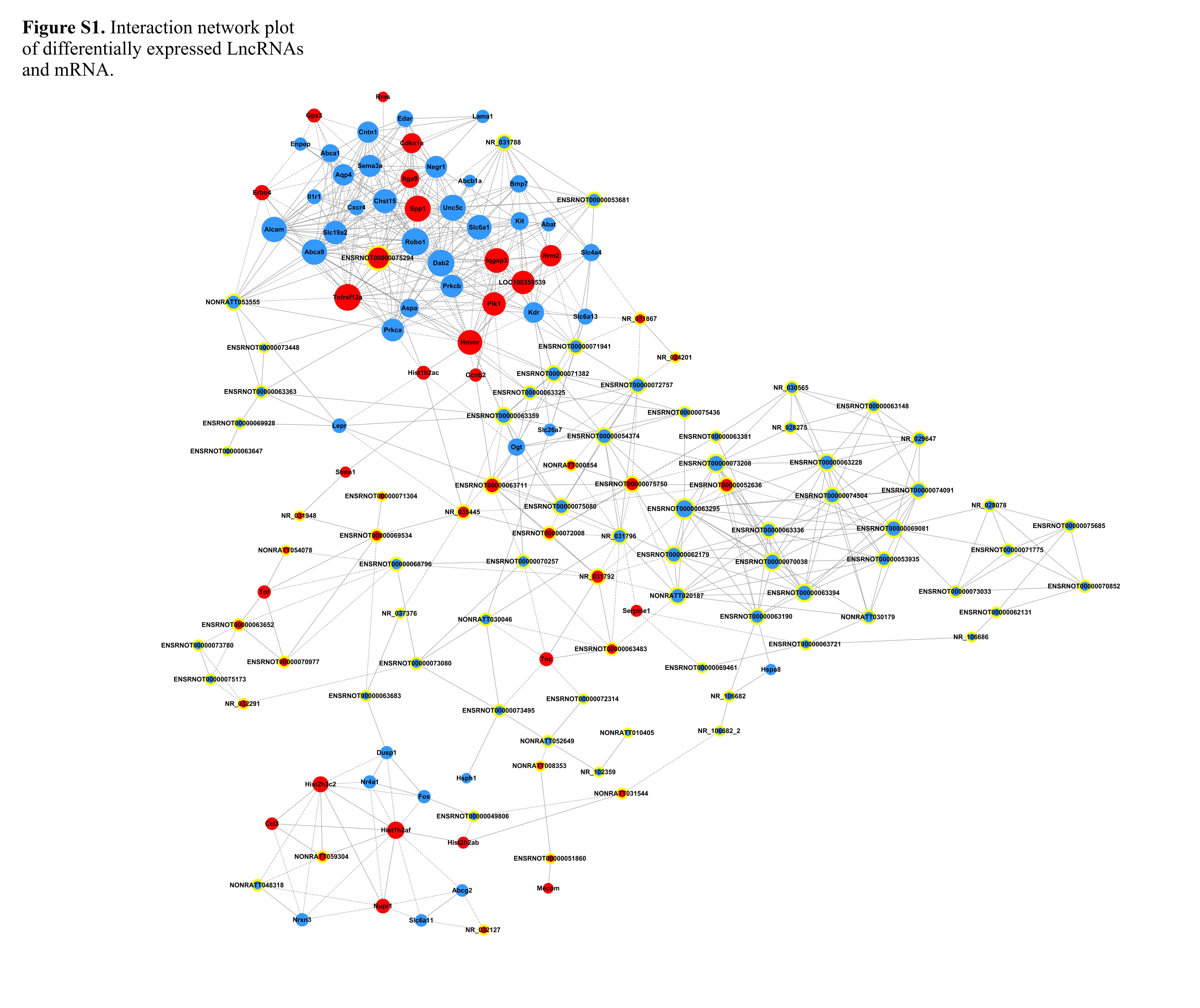

Supplement: Supplementary file 1 [file ijms-24-14531-s001.zip › Supplementary Figure S1.png]
